# Three-dimensional motion of the patella in French bulldogs with and without medial patellar luxation

**DOI:** 10.1186/s12917-021-02787-z

**Published:** 2021-02-12

**Authors:** Silvia V. Lehmann, Emanuel Andrada, Roxana Taszus, Daniel Koch, Martin S. Fischer

**Affiliations:** 1 Institute of Zoology and Evolutionary Research, Friedrich-Schiller-University Jena, Jena, Germany; 2 Daniel Koch Kleintierchirurgie AG, Diessenhofen, Switzerland

**Keywords:** Dog locomotion, Scientific Rotoscoping, Patellar motion, Patellar luxation, Three-dimensional kinematics

## Abstract

**Background:**

French bulldogs exhibit significantly larger femoral external rotation and abduction than other breeds. We were curious as to whether this peculiar leg kinematic affects patellar motion and/or might induce medial patellar subluxation (MPSL) or medial patellar permanent luxation (MPPL). We hypothesized that the more abducted leg posture during stance causes an unusual medial pull direction of the rectus femoris muscle during stance, and that this may facilitate the occurrence of MPSL or even MPPL during locomotion. To test our hypothesis, we analyzed existing stifle-joint X-ray-sequences collected during the treadmill walk and trot of seven adult female French bulldogs. We estimated 3D-patellar kinematics using Scientific Rotoscoping.

**Results:**

The three-dimensional motion of the patella comprises rotations and translations. From the seven dogs analyzed, three exhibited MPSL and one MPPL during the gait cycle. Medial patellar luxation (MPL) occurred mostly around toe-off in both gaits studied.

Patellar position was generally not gait-related at the analyzed timepoints. In dogs with MPL, the patella was placed significantly more distally (*p* = 0.037) at touch-down (TD) and at midswing (*p* = 0.024), and significantly more medial at midswing (*p* = 0.045) compared to dogs without MPL.

**Conclusions:**

Medial patellar luxation seems to be the consequence of the far from parasagittal position of the stifle joint during stance due to a broad trunk, and a wide pelvis. This peculiar leg orientation leads to a medial sideway pull caused by the rectus femoris muscle and the quadriceps femoris and may initiate plastic deformation of the growing femur and tibia. Thus, a way to avoid MPL could be to control breeding by selecting dogs with lean bodies and narrow pelvis. Actual breeding control programs based on the orthopedic examination are susceptible to errors. Systematic errors arise from the fact that the grading system is highly dependent on the dog’s condition and the veterinarians’ ability to perform the palpation on the stifle. Based on our results, the position of the patella at TD, or even perhaps during stand might offer a possibility of an objective radioscopic diagnostic of the MPL.

**Supplementary Information:**

The online version contains supplementary material available at 10.1186/s12917-021-02787-z.

## Background

The patella in dogs is a sesamoid bone that has an oval form with a non-negligible depth. Its function is generally poorly understood. It is said to increase the tension on the quadriceps femoris. Herzmark explained it using following sentence: “This principle is used in the violin to increase the tension of the strings, the bridge corresponding to the patella” [1]. It is also said that the leverage of the *musculus quadriceps femoris* is altered and increased, because the torque about the joint depends on the muscle force and the distance to the joint’s center of rotation, so that increased leverage would reduce the necessary muscle force [2]. However, it is a somewhat more complicated than that. If one takes the mechanical advantage of the knee into account, which can be described as the fraction between the lever arm of the extensor tendon to the patellar tendon [3], then the patella works as an idler gear [4]. Thus, as power is transferred between femur and tibia, the mechanical advantage amplifies not only force but also joint angular velocity [4]. In humans the mechanical advantage of the knee remains mostly lower than 1 during walking and running [5–7]. This indicates that flexion/extension velocity at the expense of force is being amplified. Mechanical advantage values lower than 1 were also reported for camels (0.75 [3],) and the ostrich (0.66–0.8 [8],). Higher mechanical advantage values were found for the Helmeted Guineafowl *(Numida meleagris)* [3–8]. No such data exist for dogs.

Overall, there is little information available on how pathologies affect patellar motion and overall stifle joint dynamics, and the genetics of such disease have only recently been reviewed [9]. In order to understand how stifle movement influences patellar position in dogs, and how muscular and non-muscular moments around the stifle joint are balanced, it is first necessary to better characterize normal and abnormal patellar kinematics. Only a few kinematic studies on patellar motion in dogs can be found. Patellar kinematics relative to the femur was described for six healthy Labrador retriever while sitting, walking, and trotting [10]. The study reported that patellar motion was coupled with the stifle flexion-extension and task dependent. In another study from the same group, Kim et al. reported altered patellofemoral kinematics in dogs with and without cranial cruciate ligament (CCL) insufficiency [11]. The study was restricted to 2D-motions. Therefore, their results cannot be applied to abnormal motions that occur mediolaterally, as in the case of the patellar luxation (PL).

Patellar luxation is a misalignment of the patella related to the femoral groove. The patella either luxates towards medial or lateral. The cause of PL is not fully understood, but several anatomic derangements such varus position of the hindlimb or the distal femur, valgus position of the proximal tibia, shallow trochlear groove, underdeveloped distal femoral condyles and torsional abnormalities have an impact or could be the sequelae of one of these [12–15]. PL was originally classified into 4 grades [16, 17] and was later redefined [18]. Grading does not necessarily correlate to the clinical presentation, which is dominated by intermittent lameness [14, 19, 20], pain on palpation, slight joint effusion, slow development of osteoarthritis and cartilage eburnation. The clinically based classification scheme could not be linked to the skeletal conformation [21–23]. Chronic medial PL can lead to ruptures of the cranial cruciate ligament [14, 24, 25].

Among small breed dogs, the medial patellar luxation (MPL) is one of the most frequent stifle pathologies [21, 24, 26, 27]. Heritability was estimated in a population of Kooiker dogs at 0.27 [28] and in Pomeranians at 0.44 [29]. It is assumed that other breeds behave in a similar way. French bulldogs especially are often affected by MPL. In a previous study, we showed that this breed exhibits significantly larger femoral external rotation and abduction than other breeds [30]. We were curious as to whether this peculiar leg kinematic affects patellar motion and/or might induce MPL. In the present work we analyzed three-dimensional kinematics of the patella in French bulldogs during walk and trot. We hypothesized that the more abducted leg posture during stance causes an unusual medial pull direction of the rectus femoris muscle during stance, and that this may facilitate the occurrence of MPL during locomotion.

## Results

**Fig. 1:**
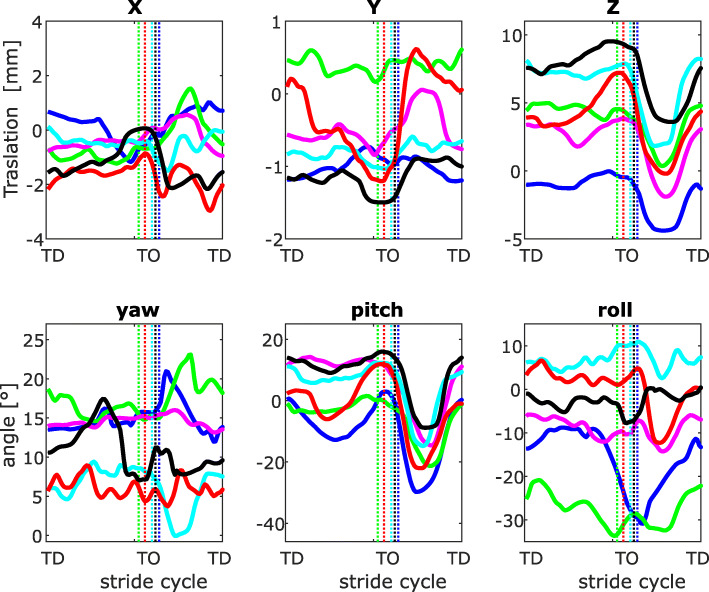
Three-dimensional kinematics of the patella with and without patellar luxation at walk. Motions are expressed related to the femur (See Fig. 6). Three-dimensional rotations and translations of the patella with respect to the center of rotation in the trochlear groove. Each curve represents the mean of the strides analyzed per dog (blue: dog 1, bright green: dog 2, magenta: dog 3, cyan: dog 4, red: dog 5, black: dog 6). Vertical dotted lines indicate toe-off

**Fig. 2:**
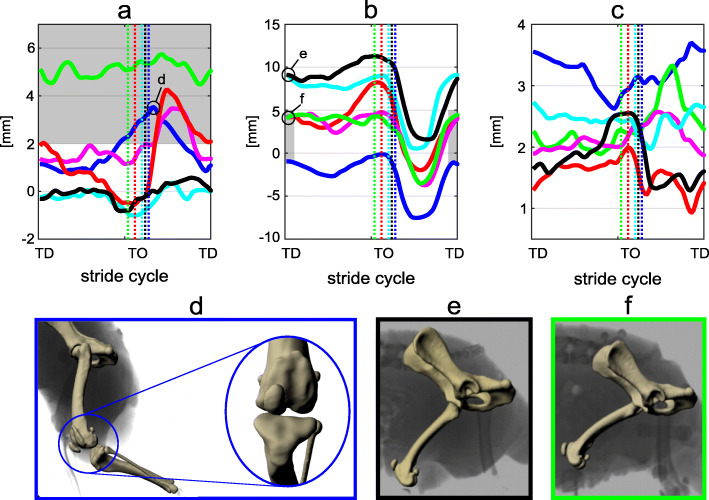
3D patellar motion at walk. **a**) sum between the patellar translation due to the rotation around the Z axis and the translation along the Y axis. **b**) sum between the patellar translation due to the rotation about the Y axis and the translation along the Z axis. **c**) sum between the patellar translation due to the rotation about the X axis and the translation along the Y axis. Renderings d, e, f based on scientific rotoscoping at selected timepoints. **d**) maximal MPL after TO. **e**, **f**) Distal-proximal position of the patella at TD. Each curve represents the mean of the strides analyzed per dog (blue: dog 1, bright green: dog 2, magenta: dog 3, cyan: dog 4, red: dog 5, black: dog 6). Vertical dotted lines indicate toe-off. Note that the patella is more distally placed in the dog with MPL. Black and cyan lines correspond to dogs without patellar luxation. Red, magenta, and blue lines correspond to dogs with MPSL. The bright green line corresponds to the dog with MPPL

**Fig. 3:**
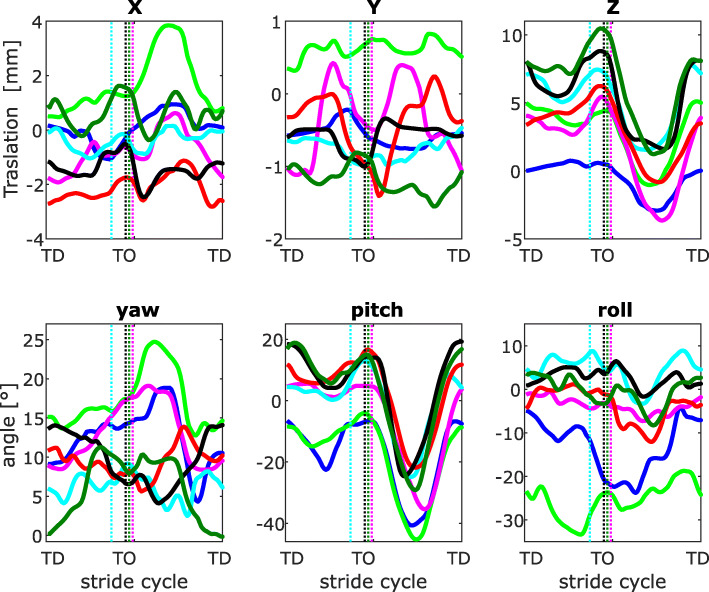
Three-dimensional kinematics of the patella with and without patellar luxation at trot. Motions are expressed related to the femur (See Fig. 6). Three-dimensional rotations and translations of the patella with respect to the center of rotation in the trochlear groove. Each curve represents the mean of the strides analyzed per dog (blue: dog 1, bright green: dog 2, magenta: dog 3, cyan: dog 4, red: dog 5, black: dog 6, dark green: dog 7). Vertical dotted lines indicate toe-off

**Fig. 4:**
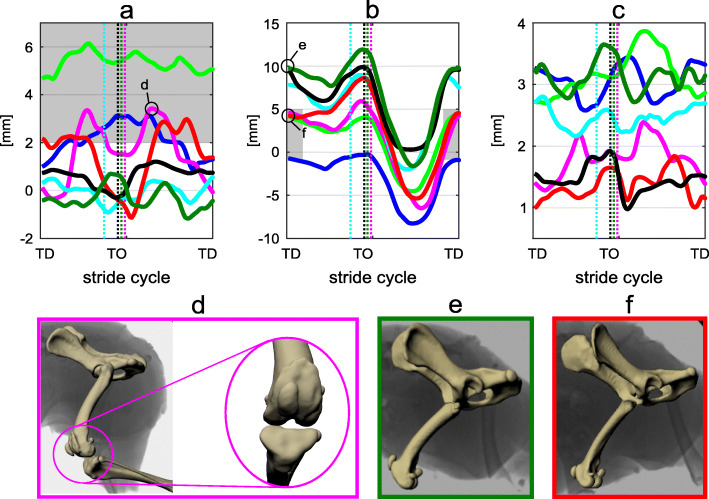
3D patellar motion at trot. **a**) sum between the patellar translation due to the rotation around the Z axis and the translation along the Y axis. **b**) sum between the patellar translation due to the rotation about the Y axis and the translation along the Z axis. **c**) sum between the patellar translation due to the rotation about the X axis and the translation along the Y axis. Renderings d, e, f based on scientific rotoscoping at selected time-points. **d**) maximal MPL after TO. **e**, **f**) Distal-proximal position of the patella at TD. Each curve represents the mean of the strides analyzed per dog (blue: dog 1, bright green: dog 2, magenta: dog 3, cyan: dog 4, red: dog 5, black: dog 6, dark green: dog 7). Vertical dotted lines indicate toe-off. Note that the patella is more distally placed in the dog with MPL. Black, dark green, and cyan lines correspond to dogs without patellar luxation. Red, magenta, and blue lines correspond to dogs with MPSL. The bright green line corresponds to the dog with MPPL

We analyzed walking data from six bulldogs and trot data from seven bulldogs. In the walk videos from dog number 7, the patella was out the field of view during larger parts of the stride, so that the rotoscoping of the patella was not possible. The motion of the patella comprises rotations and translations (Figs. 1, 3). The sum of both motions expresses the actual position of the patella (Figs. 2, 4). We found MPL in four of the seven dogs occurring during the gait cycle. These four dogs did not display any sign of pain or incommodity (e.g., sign of stress, such as pants, non-periodic or abnormal movement patterns or lameness) during locomotion tasks. Three of them exhibited medial patellar subluxation (MPSL) and one exhibited medial patellar permanent luxation (MPPL).

Patellar position (sum of rotations and translations related to the patellar joint at an instant time) is generally not gait-related at the analyzed timepoints. Exceptions are the lateromedial (LM) position at midstance and the distal-proximal (DP) position at midswing (*p* = 0.003, respectively *p* = 0.012). The distal-proximal and the LM position of the patella were found to be significantly different at touch-down (TD) (DP: *p* = 0.037) and at midswing (DP: *p* = 0.024, LM: *p* = 0.045) between dogs with and without MPL. The product MPL by gait was only significant for the LM and DP displacements both at midstance (LM: *p* = 0.017, DP: 0.046). The contrast analyses for DP motions between dogs with and without MPL show: (1) at TD, differences are significant for walk and trot; (2) at midstance, significant differences were found only for walk; (3) at midswing, differences are significant for walk and trot. The contrast analyses for LM motions between dogs with and without MPL depicted significant differences at midswing only for walk (see Table 1).

**Table 1:** Mean, standard deviations of patellar movements, and results of repeated measures ANOVA and contrast analysis. Data from 6 French bulldogs (2 without and 4 with Medial Patellar Luxation) in the distal-proximal, latero-medial, and craniocaudal axes

	MPL	% Stance		% Swing	
		TD	50	TO	50
**Latero-Medial displacement**					
Walk	0	−0.3 ± 0.0	0.0 ± 0.0	− 0.6 ± 0.5	0.3 ± 0.2*
	1	2.4 ± 1.9	2.1 ± 1.8	2.5 ± 2.4	3.7 ± 1.4*
Trot	0	0.5 ± 0.3	0.3 ± 0.4	− 0.5 ± 0.4	0.7 ± 0.5
	1	2.0 ± 2.0	3.3 ± 1.8	2.4 ± 2.4	3.2 ± 1.3
Gait		n.s.	***p*** **= 0.003**	n.s.	n.s.
MPL		n.s.	n.s.	n.s.	***p*** **= 0.045**
Gait*MPL		n.s.	***p*** **= 0.017**	n.s.	*p* = 0.057
**Distal-Proximal displacement**					
Walk	0	9.1 ± 0.0*	8.5 ± 1.2*	9.5 ± 1.4	1.2 ± 0.6*
	1	2.9 ± 2.6*	2.0 ± 2.9*	3.6 ± 4.2	−4.0 ± 2.2*
Trot	0	8.6 ± 1.2*	5.7 ± 0.5	8.9 ± 1.3	−0.8 ± 1.6*
	1	3.1 ± 2.5*	2.0 ± 2.6	4.2 ± 3.7	−6.0 ± 1.6*
Gait		n.s.	*p* = 0.052	n.s.	***p*** **= 0.012**
MPL		***p*** **= 0.037**	*p* = 0.066	n.s.	***p*** **= 0.024**
Gait*MPL		n.s.	***p*** **= 0.046**	n.s.	n.s.
**Craniocaudal displacement**					
Walk	0	4.4 ± 1.5	4.3 ± 0.9	4.7 ± 0.1	3.9 ± 1.3
	1	4.5 ± 1.9	4.6 ± 1.3	4.9 ± 0.9	5.2 ± 1.6
Trot	0	4.3 ± 1.6	3.7 ± 1.2	4.4 ± 0.8	3.9 ± 1.6
	1	4.2 ± 2.2	4.6 ± 1.7	5.0 ± 1.7	5.0 ± 1.9
Gait		n.s.	n.s.	n.s.	n.s.
MPL		n.s.	n.s.	n.s.	n.s.
Gait*MPL		n.s.	n.s.	n.s.	n.s.

Displacements are presented in millimeters [mm]. TD: touch-down, TO: toe-off. 0 = Dogs without MPL, 1 = Dogs with MPL. Values displayed in bold indicate that the differences are significant (*p < 0.05). n.s. = non-significant

### Description of patellar kinematics at walk

#### 3D patellar translations

During stance translations in x (cranio-caudal) direction oscillates between − 2 mm and 0.5 mm and display no clear differences between dogs with and without PL. After toe-off (TO) the patella moves cranially in two dogs and caudally in the other four. In the late swing phase translation turns towards the values observed at TD (Fig. 1 upper left).

Translations in the y (medio-lateral) direction oscillate between 0 and 3 mm. During stance, two of the four dogs with MPL exhibited mediolateral translations larger than 2 mm. The patella of the dog with MPPL remains during stance and swing phases 3 mm more medially with respect to the center of the trochlear groove. During swing, two of the three dogs with MPSL exhibited mediolateral translations larger than 2 mm.

Translations in z (DP) direction display differences between dogs with and without PL. At TD, dogs with MPL held their patella more distally (around 5 mm) than in healthy dogs (around 7.5 mm). The patella remains in this position during approx. 50% of the stance, before it starts to move proximally till TO. After TO the patella moves distally until midswing and then returns to the values of TD. Patellar rotations are more pronounced during the swing phase.

#### 3D patellar rotations

At TD the patella is rotated externally between 5° and 17° (rotations around x = yaw). With the exception of one dog, which increased external rotation after TD, the orientation of the patella related to the x-axis did not significantly change during stance. After TO rotations of the patella did not display a general pattern. In most of the analyzed dogs, the patellar external rotation was increased by approx. 5°, and then decreased to the values observed at TD.

Rotations around y (pitch) display a general pattern among all dogs. Its pattern resembles the translations in z, as the patella moved distally (pitch angle) during the first half of the stance phase. After midstance, the patella returns to its position observed in TD. After TO, the patella moves distally approx. 20°, and then returns to the TD position from midswing on.

Rotations around z (see “roll,” Fig. 1, bottom right) did not displayed significant variations during stance. Only the dog with MPPL displayed larger angles than − 20°, the other dogs displayed roll angles between 5° and − 10° (negative angles denote rotations towards medial). After TO, dogs with MPL displayed rapid medial patellar rotations. After a maximum rotation, which varied between 10° and 25°, the patella returned to the orientation previously observed at TD.

#### 3D patellar position

Figure 2 shows the sum of the combined translations and rotations at walk. The distance along the medio-lateral axis between the center of the patella and the center of the trochlear groove is shown in Fig. 2a. For the two dogs without MPL, the position of the patella remains close to the center of the trochlear groove. The three dogs with MPSL displayed peak amplitudes larger than 3 mm towards medial after TO. The center of the patella in the dog with MPPL remained approx. 5 mm medially displaced during both stance and swing phase. Figure 2b introduces the distance along the proximo-distal axis between the center of the patella and the center of the patella-femoral joint. This figure displays small discrepancies related to the translations in z. Here it is also clear that at TD, and during the early stance dogs with MPL held their patella (significantly, *p* < 0.05) more distally (bellow 5 mm) than did the healthy dogs (around 9 mm). Figure 2c presents the craniocaudal position of the patella during a stride. No clear motion patterns or differences between dogs with and without MPL could be observed.

### Description of patellar kinematics at trot

#### 3D patellar translations

During stance‚ translations in x (craniocaudal) direction oscillates between − 3 mm and 4 mm. The last amplitude was only exhibited by the dog with MPPL at midswing. No clear differences could be observed between dogs with MPSL and without MPL. After TO, the patella moves cranially and caudally alternatively. In the late swing phase, translation turns towards the values observed at TD (Fig. 3 upper left).

Translations in y (medio-lateral) direction oscillate between − 1 and 3.5 mm. During stance, three of the four dogs with MPL exhibited mediolateral translations larger than 2 mm. The patella of the dog with MPPL remained during stance and swing phases approx. 3.5 mm more medially with respect to the center of the trochlear groove. During swing, two of the three dogs with MPSL exhibited mediolateral translations larger than 2 mm.

Translations in z (distal-proximal) direction are quite similar to those exhibited at walk.

#### 3D patellar rotations

At TD, the patella is rotated externally between 0° and 25° (rotations around x = yaw). After TD, rotations of the patella did not display a general pattern. After TO, three of the four dogs with MPL exhibited the largest yaw rotations.

Rotations around y (pitch) display the same general pattern as observed in walk, but with larger amplitudes around midswing.

Rotations around z (see “roll,” Fig. 3 bottom right) were similar to those already explained for walk.

#### 3D patellar position

Figure 4 shows the sum of the combined translations and rotations for trot. The distance along the medio-lateral axis between the center of the patella and the center of the trochlear groove is shown in Fig. 4a. For the three dogs without MPL, the position of the patella remained close to the center of the trochlear groove. The three dogs with MPSL displayed peaks amplitudes larger than 3 mm towards medial after TO. Two of them even displayed MPL in the late stance phase. The time-point of the maximal MPL differs between dogs. This fact precluded the finding of the significant differences between dogs with and without MPL. Still, during walk we found significant differences at midswing. The center of the patella in the dog with MPPL stayed approx. 5 mm medially displaced during both stance and swing phase. Figure 4b introduces the distance along the proximo-distal axis between the center of the patella and the center of the patella-femoral joint. Here it is clear that at TD and at midswing, dogs with MPL held their patella significantly (*p* < 0.05) more distally. Figure 4c presents the craniocaudal position of the patella during a stride. As observed at walk, no patterns or differences between dogs with and without MPL exist.

## Discussion

This is the first description of the three-dimensional kinematics of patella motion of one dog breed at walk and trot based on biplanar high-speed fluoroscopy. Comparison with other dog breeds is still lacking. The selection of French bulldogs is based on previous findings of an unusual 3D-kinematic in the hindlimb of this breed as well as the unique possibility to analyze dogs with and without medial patellar luxation [30].

French bulldogs display surprisingly larger femoral abduction (> 40°) and external rotation (> 30°) during walk and trot [30]. This fact set them apart from the healthy breeds studied so far [e.g. [30–35],]. Interestingly, stifle torsion in French bulldogs is not significantly different from those observed in other breeds. Thus, the extreme abduction in French bulldogs is maintained throughout the whole limb during stance, while the femoral axial rotation induces larger pelvic medial translations [30].

Two-dimensional patellar kinematics in the dog showed that the motion of the patella relative to the femur is coupled with the stifle flexion-extension and task related [10]. In addition, the patella was also found to be more distally placed in dogs with CCL insufficiency [11].

Stylianou and colleagues used a cadaveric pelvic limb of an adult dog to analyze stifle joint (including patellar) kinematics and forces. In their experiment, the authors displaced the pelvis vertically 75 mm, producing a flexion of the stifle. Such a vertical excursion exceeds by far those we estimated in our scientific rotoscoping studies at walk or trot (e.g., French bulldogs approx. 15 mm, Labradors approx. 20 mm). A comparison is further hampered because the authors presented their results in a way that makes quite difficult to read the amplitude of the motions [36].

### Medial patellar luxation (MPL)

Medial patellar luxation occurs mostly around TO in both gaits studied. In dogs with MPSL, the patella moves medially approx. 35% of its width. This value increased to more than 60% in the dog with MPPL. Dogs with MPL exhibited a more distally placed position of the patella at TD, and sometimes reduced vertical motion. During walk, dogs displayed similar patellar patterns as in trot, however, the amplitude of the motions was lower. Dogs exhibited the same MPL grade as in trot.

It has been suggested that MPSL is related to tibial torsion (known as the “screw-home” mechanism) during the early swing phase [14, 24]. We do not agree with this view as our previous study has shown that the femur undergoes not only a significant abduction of up to 30°, but also an external rotation in about the same magnitude during stance in the walking and trotting French bulldog [30]. It is the consequence of this far from parasagittal position of the stifle joint and so the femoropatellar joint which leads to a medial sideway pull caused by the unusual direction of the rectus femoris muscle and the quadriceps femoris angle. Similar ideas are the basis of realignment of the quadriceps mechanism with distal femoral corrective osteotomy [37].

The more distally placed position of the patella at TD found in dogs with MPL could indicate that either the *ligamentum patellae* has been shortened, or that the tendon of the quadriceps femoris muscle has been elongated, for which the last-mentioned idea seems more plausible of both.

A hypothesis to be tested is that MPL relates to a broad trunk, a wide pelvis, and hence the distance between feet at touch-down or the width of foot trajectories. The greater the width the more abducted and externally rotated will be the limb kinematics. Body weight, or better overweight, might have a crucial impact on this. The quadriceps femoris muscle as anti-gravity muscle will pull on the patella in an oblique direction to the femur, especially after midstance. Depending on the relative distance between midpoint of the body and touch-down point, this angulation results in medial pull, as well as on the patella and secondary rolling of the femur. Muscle forces then by time force the patella out of the sulcus, resulting in PL. Whether this relates in any way to the 12 times more common MPL in small dogs remains open.

Knowledge of patellar kinematics and dynamics may open alternative pathways for a better understanding of the pathogenesis of PL and open new possibilities for classifying. The impact on surgical treatment, however, remains open and necessitates further clinical research.

Regarding pathogenesis, our findings place the rolling mechanism of the femur in a central role, attributing it an initiating mechanism to many other and well described accompanying findings. By opening a wider anatomical and biomechanical frame for the understanding of MPL, the pathogenetic explanation starts with a broad and tilting pelvis, which leads to a rolling and abduction of the femur in the stance phase, not allowing the patella to follow its movement because it is held in position by the fixed lower hind limb. As these peculiar movement pattern already starts early in the life of the dog, the weak bones of the growing animal must undergo plastic deformation by strong muscle pull. These plastic deformations however are well known, summarized as malalignment of the pelvic limb and specifically described as coxa vara, genu varum, distal femora varisation, proximal tibial valgisation, torsional deformity of the near stifle region and shallowing of the femoral trochlear groove [12, 13, 38]. And further on, the wide ranged, dynamically and muscle force driven explanation for MPL could be the reason, why the grading of MPL could not the correlated to the rather static skeleton in the vicinity of the stifle joint [21–23]. In order to substantiate the new formulated explanation for MPL, we suggest a longitudinal anatomical and radiological study with puppies from predisposed breeds.

Up to now, breeding control programs were based on the orthopedic examination, and the grading system established in the late 1960s [16, 17]. However, this rather subjective evaluation is susceptible to errors [19], and did not result in a reliable improvement of the canine breeds known to have MPL [39]. Systematic errors arise from the fact that the grading system is highly dependent on the dog’s condition and the veterinarians’ ability to perform the palpation on the stifle. More reliability would result from a method, whereby undoubtable facts could be measured or assessed by different persons or by machines. Such standards exist for the use of radiographs in the hip dysplasia and elbow dysplasia programs or by blood examinations. Based on our results, the position of the patella at TD, or even perhaps during stand might offer such a possibility. However, a higher cohort of dogs is necessary to test this hypothesis.

An alternative way to avoid MPL could be to control breeding by selecting dogs with lean bodies and narrow pelvis. Support for this theory comes from a historical point of view. The patella is nowadays considered as a sesamoid bone, but as a remnant of an apophysis at the distal femur. The rectus femoris muscle found its insertion there, leaving the patellar ligament a quite stiff cranial stifle stabilizer. Other parts of the quadriceps muscle ended in the medial and lateral fascia inserting at the pes anserinus on the medial tibia and on the fascia of the cranial tibial muscle. Detaching the apophysis from the femur resulted in free moveable bone, the patella, which then came under the influence of laterally and medially located muscles, which controlled adduction and abduction. If the medial vastus muscle now pulls towards medial at the same time as the femur abducts, as in the case of broad stance or high body weight, the patella luxates.

## Conclusion

Medial patellar luxation occurs mostly around TO in both gaits studied. It seems to be the consequence of the far from parasagittal position of the stifle joint during stance. This peculiar leg orientation leads to a medial sideway pull caused by the rectus femoris muscle of the quadriceps femoris. Thus, the pelvis conformation is a possible indicator for a MPL. Further research on the form of the pelvis, pelvic width, and acetabular retroversion is required to support this theory, and to establish a possible new standard for breeding control programs. It must be noted, that even when correlation or the clinical occurrence of MPL could be found, the trigger to MPL could be somewhere else, and pelvis conformation is only the result of a different or remote trigger. Finally, our results showed that the position of the patella at TD, or even perhaps during stand might offer the possibility of radioscopic diagnostic of the MPL.

## Methods

**Fig. 5:**
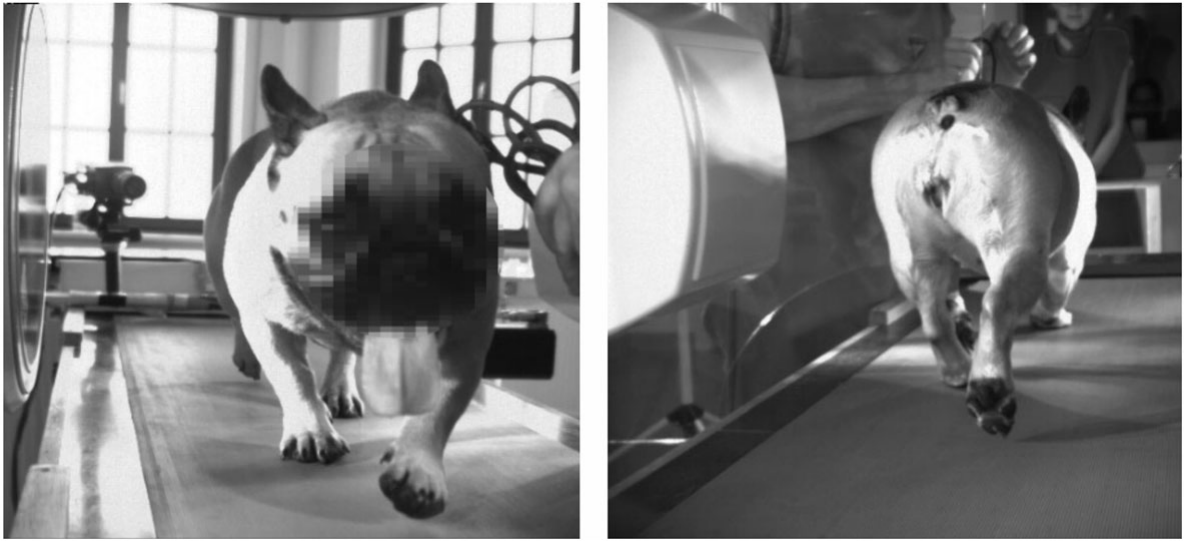
French bulldog trotting on a treadmill in the X-ray-Lab

We selected seven adult female French bulldogs data from a pool of dogs who participated to earlier studies [four dogs belonged to [30] and another three to a not published study to trunk kinematics, see acknowledgements and Table 2]. In those studies, the owners have been asked whether any of their dogs has had orthopedic problems or have ever been treated in this way. The dogs were visually examined by a veterinarian during walking. Only dogs with absence of clinical signs were included in those studies. We analyzed the stifle-joint X-ray-sequences obtained during the treadmill walk and trot, focusing on patellar motion (Fig. 5).

**Table 2:** Information about dogs, patella, and trochlea dimensions, and analyzed data

Dog Number	Dog Name	Age [years]	Weight [kg]	Height at the withers [mm]	Patella length [mm] A	Patella width [mm] B	Patella depth [mm] C	Trochlea width [mm] D	Trochlea depth [mm] E	Leg	MPL	Gait	Speed [m/s]	Strides
1	Qeny	7	11	310	14.4	8.2	6.1	7.4	^a^	L	1	w	0.7	10
										L	1	t	1.3	8
2	MJ	7	9.5	300	CT not available	CT not available	CT not available	CT not available	CT not available	R	3	w	0.7	3
										R	3	t	1.7	10
3	Chacha	7	10	310	CT not available	CT not available	CT not available	CT not available	CT not available	L	1	w	0.6	8
										L	1	t	1.2	8
4	Juno	2	13	320	CT not available	CT not available	CT not available	CT not available	CT not available	L	0	w	0.7	10
										L	0	t	1.5	6
5	Luna	3	9.5	280	12.0	8.0	5.7	7.9	1.1	L(^c^)	1	w	0.8	10
					11.7	8.0	5.8	7.9	1.1	R	1	t	1.1	9
6	Esprit	1	–	325	CT not available	CT not available	CT not available	CT not available	CT not available	R	0	w	0.7	5
										R	0	t	1.2	10
7	Gretel	3	13.5	330	14.7	9.1	6.6	8.8	1.7	R (^b^)	0	t	1.3	9

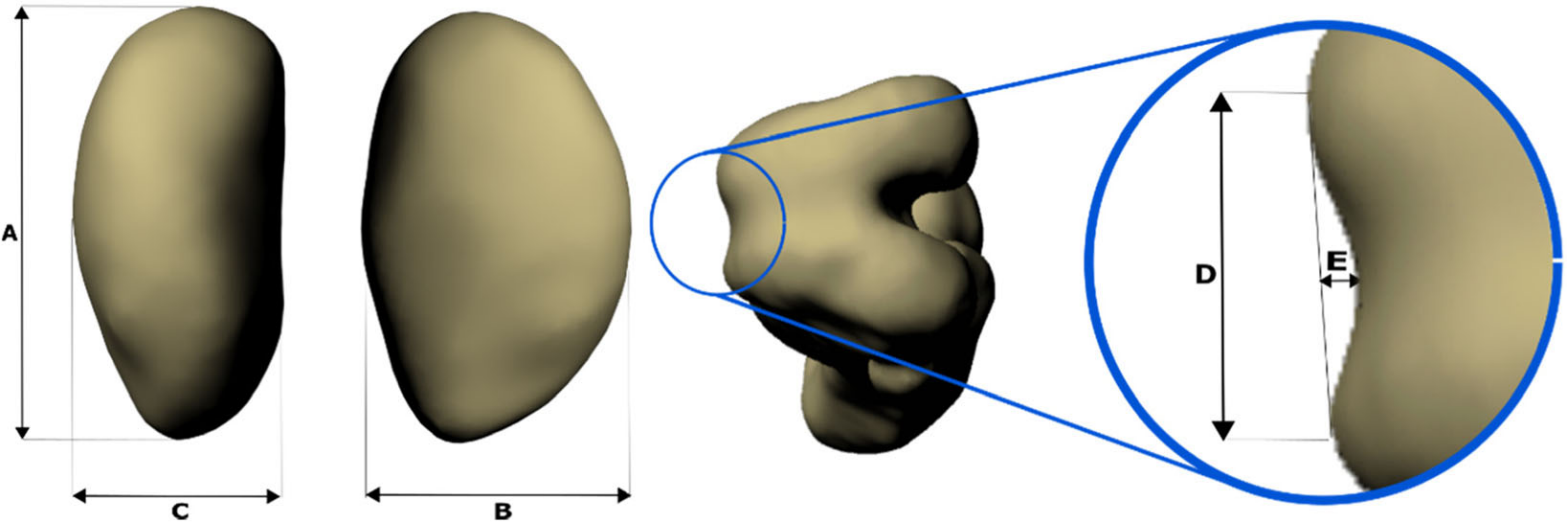
*L* Left, *R* Right, *w* walk, *t* Trot

*MPL* Medial Patellar Luxation, 0 = no MPL, 1 = Grad 1, 3 = Grad 3. Height at the withers was measured while the dogs were standing. ^a^Trochlea severely worn, the trochlea depth could not be measured. ^b^ virtual reconstruction of the patella belongs to this dog. ^c^virtual reconstruction of the femur belongs to this dog. Dogs 1, 2, 3, 4 belonged to [30], while 5, 6, and 7 to a not published study to trunk kinematics, see acknowledgements

As the dogs were presented as clinically sound, no clinically based grading is available. We therefore describe it as healthy condition, when the patella remains within the trochlear groove (*facies patellaris*), using MPSL to describe a motion when the patella moves from the trochlea to the condylar ridge and remains there for a certain period, and MPPL when the patella is permanently on the exterior medial surface of the trochlea ridge.

To reconstruct 3D-kinematics we used the method known as Scientific Rotoscoping [see [30] for an extended description of the methodology]. For this we use the Autodesk Maya™ software with the XROMM Maya Tools (www.xromm.org; Brown University, Providence, RI, US).

In this study, our 3D model is composed of three segments: pelvis, femur, and patella. Virtual bones were obtained from the virtual CT reconstruction (Amira 6.3.0, Thermo Fisher Scientific). We limited ourselves to three whole-body CT scans. For the dogs 2, 3, 4, and 6, we have used scaled skeletal elements in Maya™. Note that in Autodesk Maya™, movements are measured in relation to a reference pose. We based the construction of our reference pose on the methods presented in [30]. Accordingly, in all joints of the reference pose, +x points caudally, +y points medially and + z upwards. Hip and knee joints were aligned in the frontal and sagittal planes. 3D joint angles were computed according to the rotation sequence y, x, z, (i.e.: pro/retraction, ad/abduction, axial rotation) [30].

**Fig. 6:**
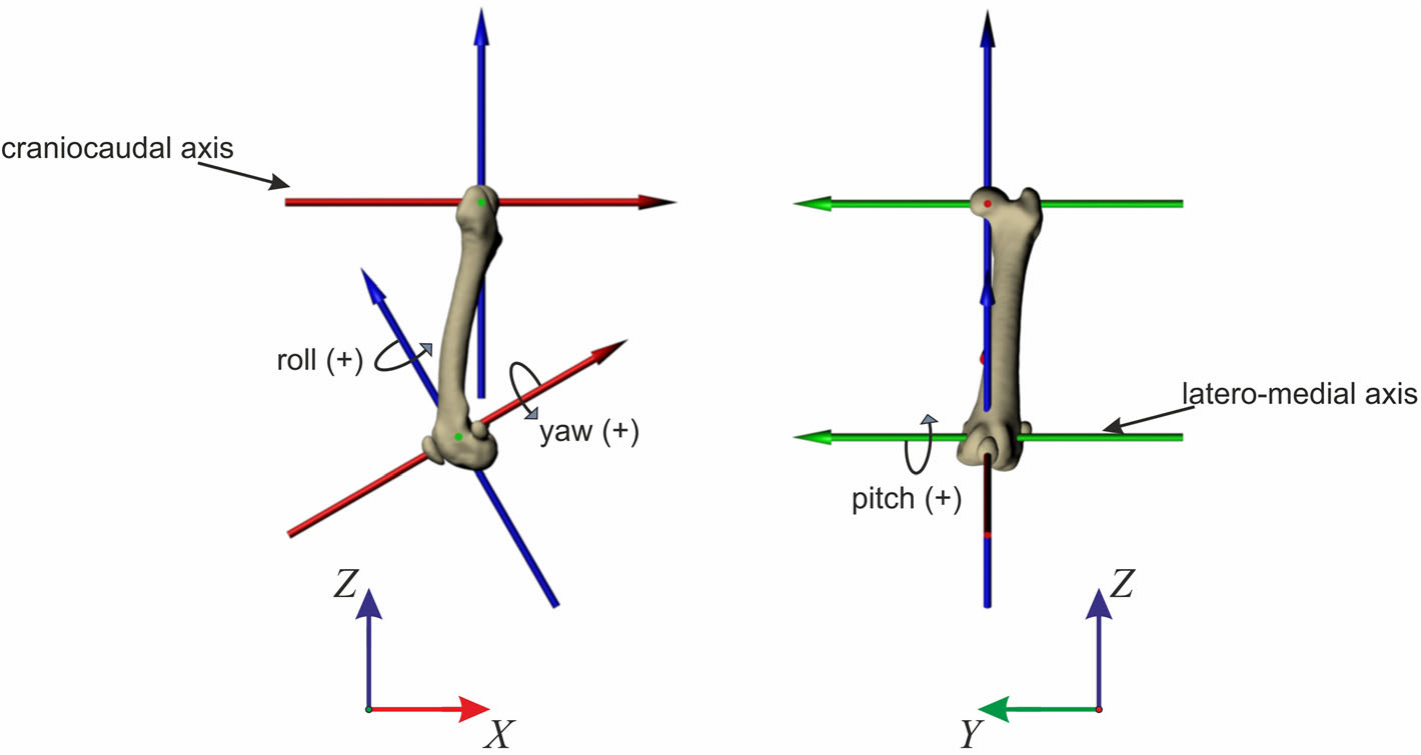
Zero-pose and axes for the patella. Patellar motions were measured relative to this pose. Every dog has its own reference pose. However, all models were build following the same kinematic chain. Joint coordinate systems were aligned to the axes of the global coordinate system (+x points caudally, +y points medially and + z upwards). Note that the patella was rotated 30° around the Y-axis relative to the patellofemoral joint. Green, blue, and red dots indicate that those axes point into the picture

The coordinate systems of the model reference pose were located in: (1) the middle of the pelvis, (2) in the hip joint, and (3) in the patellofemoral joint. They permit to measure the motion of the pelvis related to global coordinate system, femoral movement relative to the pelvis, and the patellar movement relative to the femur, respectively. Both pelvic and hip joint coordinate systems were located following the method explained in [30]. The patellofemoral joint was located in the middle of a cube that encompassed the form of the distal femur (see Fig. S1). The zero position of the patella was set as follows: A sphere was centered in the middle of the patellofemoral joint. Its radius was adjusted until its surface touched the surface of the *Facies patellaris ossis femoris* (see Fig. S2). The coordinate system for the patella was located in the center of the sphere, in such a way that the X axis, which represents the movements of ad−/abduction, passes between both *condyli*. The patella was placed in vertical position, with the *basis patellae* and the *apex patellae* approximately aligned in the sagittal plane. In the frontal plane, the patella was centered in the sphere. Then, the patella was moved towards the sphere (in both sagittal and dorsoventral planes) until the vertical crest of the *facies articularis* of the patella touched the sphere. A cube that enclosed the patella was used to find the patella’s midpoint [ [40], see Fig. S3]. Patellar coordinate system was then aligned with the patellofemoral joint (see Fig. S4). Finally, the patella was centered in the marked rectangle of the *Facies patellaris ossis femoris* by rotating the patella 30° around the Y-axis relative to the patellofemoral joint (Fig. 6).

Pelvis, femur, and patella were manually rotated and translated in Maya™ until they matched their X-ray image in both views. This process was repeated every 10 to 25 frames. The resulting “key frames” were then interpolated using cubic splines and exported to Matlab™. In Matlab™, the exported data were normalized before computing mean and standard deviation (s.d.).

The accuracy and repeatability of the rotoscoping was estimated similarly to [30]. The manual matching at 4 fixed time-points during a stride (TD, midstance, TO, and midswing) was repeated six times. Standard deviation (SD) was estimated for the six degrees of freedom (DOF) at the patellofemoral joint. Mean SD over a stride was used as a metric to describe the matching error of the patella. On average computed position errors were less than ±0.5 mm for the translations in x, y and z. Average angular variations were rather similar for all axes and did not exceed ±2.6°.

To analyze the influence of both gait and PL on patellar kinematics we used repeated measures ANOVA. Comparisons were performed at the same specific timepoints as in [30] (TD, midstance, TO, and midswing). Gait (walk vs. trot) was set as within subjects and MPL as between subjects. Afterwards, simple contrast tests with Bonferoni correction were used to test for differences between dogs with and without MPL at each gait. Statistical analysis was performed in IBM® SPSS® Statistics 26.

## Supplementary Information

**Additional file 1: Supplementary information to the patellofemoral joint: steps to model the patellofemoral joint. Figure S1.** Location of the patellofemoral joint in the middle of a cube that encompassed the form of the distal femur. Coordinate systems were aligned to the axes of the global coordinate system [x (red, positive direction points caudally, y (green, positive direction points medially) and z (blue, positive direction points upwards)]. Blue, and red dots indicate that those axes point into the picture. **Figure S2.** The zero position of the patella was set as follows: A sphere was centered in the middle of the patellofemoral joint (see Fig. S1). Its radius was adjusted until its surface touched the surface of the *Facies patellaris ossis femoris.* **Figure S3.** The patella was placed in vertical position, with the *basis patellae* and the *apex patellae* approximately aligned in the sagittal plane. In the frontal plane, the patella was centered in the sphere (see Fig. S2). Then, the patella was moved towards the sphere (in both sagittal and dorsoventral planes) until the vertical crest of the *facies articularis* of the patella touched the sphere. A cube that enclosed the patella was used to find the patella’s midpoint.Coordinate systems were aligned to the axes of the global coordinate system [x (red, positive direction points caudally, y (green, positive direction points medially) and z (blue, positive direction points upwards)]. Green, and red dots indicate that those axes point into the picture. **Figure S4.** The vertical crest of the *facies articularis* of the patella was moved until it touched the sphere (compared to Figures S2 and S3, the femur was made invisible in this render). Patellar coordinate system was then aligned with the patellofemoral joint. Green dots indicate that those axes point into the picture.

## Data Availability

The datasets used and/or analyzed during the current study are available from the corresponding author on reasonable request.

## Abbreviations

CCL: Cranial cruciate ligament
DOF: Degree of freedom
DP: Distal-proximal
LM: Lateromedial
MPL: Medial Patellar Luxation
MPPL: Medial Patellar Permanent Luxation
MPSL: Medial Patellar Subluxation
PL: Patellar luxation
TD: Touch-down
TO: Toe-off
