# Supplementary material for: Three-dimensional motion of the patella in French bulldogs with and without medial patellar luxation
Source: BMC Vet Res. 2021 Feb 12;17:76. doi: 10.1186/s12917-021-02787-z (PMC7881552; doi:10.1186/s12917-021-02787-z)
Supplement: Supplementary file 1 — Additional file 1: Supplementary information to the patellofemoral joint: steps to model the patellofemoral joint. Figure S1. Location of the patellofemoral joint in the middle of a cube that encompassed the form of the distal femur. Coordinate systems were aligned to the axes of the global coordinate system [x (red, positive direction points caudally, y (green, positive direction points medially) and z (blue, positive direction points upwards)]. Blue, and red dots indicate that those axes point into the picture. Figure S2. The zero position of the patella was set as follows: A sphere was centered in the middle of the patellofemoral joint (see Fig. S1). Its radius was adjusted until its surface touched the surface of the Facies patellaris ossis femoris. Figure S3. The patella was placed in vertical position, with the basis patellae and the apex patellae approximately aligned in the sagittal plane. In the frontal plane, the patella was centered in the sphere (see Fig. S2). Then, the patella was moved towards the sphere (in both sagittal and dorsoventral planes) until the vertical crest of the facies articularis of the patella touched the sphere. A cube that enclosed the patella was used to find the patella’s midpoint.Coordinate systems were aligned to the axes of the global coordinate system [x (red, positive direction points caudally, y (green, positive direction points medially) and z (blue, positive direction points upwards)]. Green, and red dots indicate that those axes point into the picture. Figure S4. The vertical crest of the facies articularis of the patella was moved until it touched the sphere (compared to Figures S2 and S3, the femur was made invisible in this render). Patellar coordinate system was then aligned with the patellofemoral joint. Green dots indicate that those axes point into the picture. [file 12917_2021_2787_MOESM1_ESM.pdf]

Three-dimensional motion of the patella in French bulldogs with and without medial patellar luxation

Silvia V. Lehmann[1], Emanuel Andrada[1], Roxana Taszus[1], Daniel Koch[2], Martin S. Fischer[1]

1 Institute of Zoology and Evolutionary Research, Friedrich-Schiller-University Jena, Germany;

2 Daniel Koch Kleintierchirurgie AG, Diessenhofen, Switzerland.

Supplementary information to the patellofemoral joint

Steps to model the patellofemoral joint.

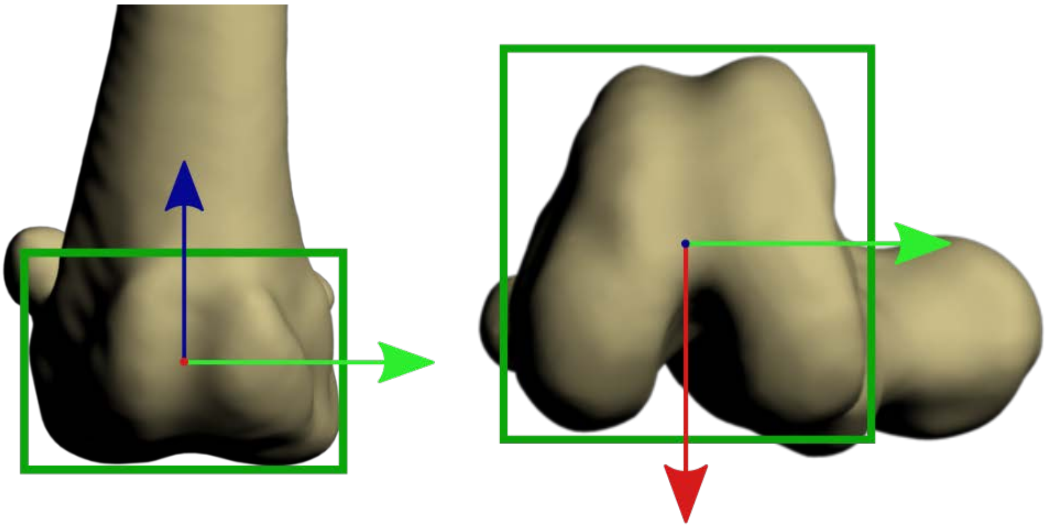

Figure S1 Location of the patellofemoral joint in the middle of a cube that encompassed the form of the distal femur. Coordinate systems were aligned to the axes of the global coordinate system [x (red, positive direction points caudally, y (green, positive direction points medially) and z (blue, positive direction points upwards)]. Blue, and red dots indicate that those axes point into the picture.

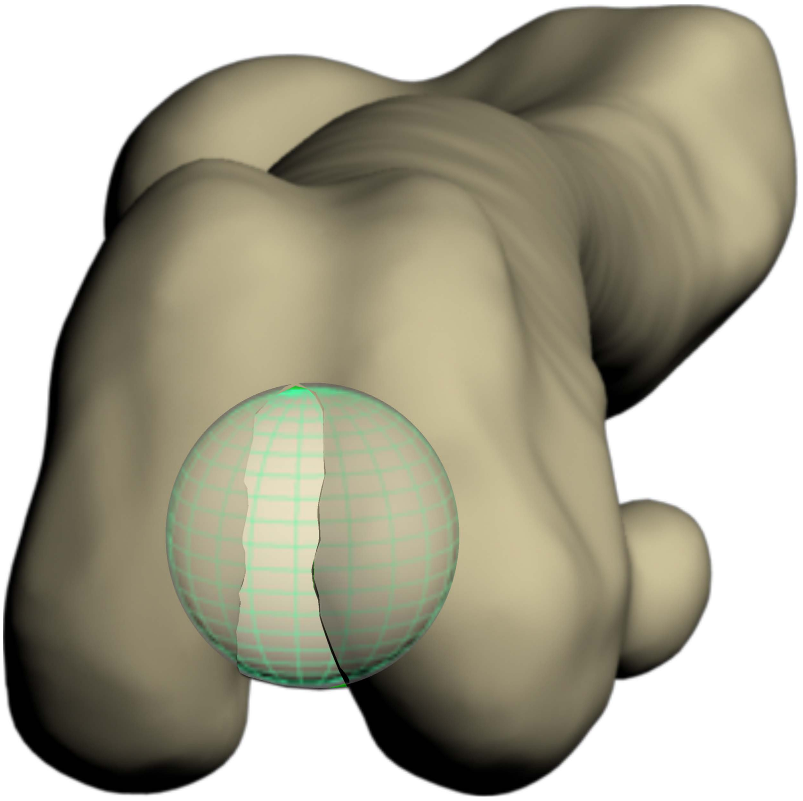

Figure S2 The zero position of the patella was set as follows: A sphere was centered in the middle of the patellofemoral joint (see Fig. S1). Its radius was adjusted until its surface touched the surface of the *Facies patellaris ossis femoris.*

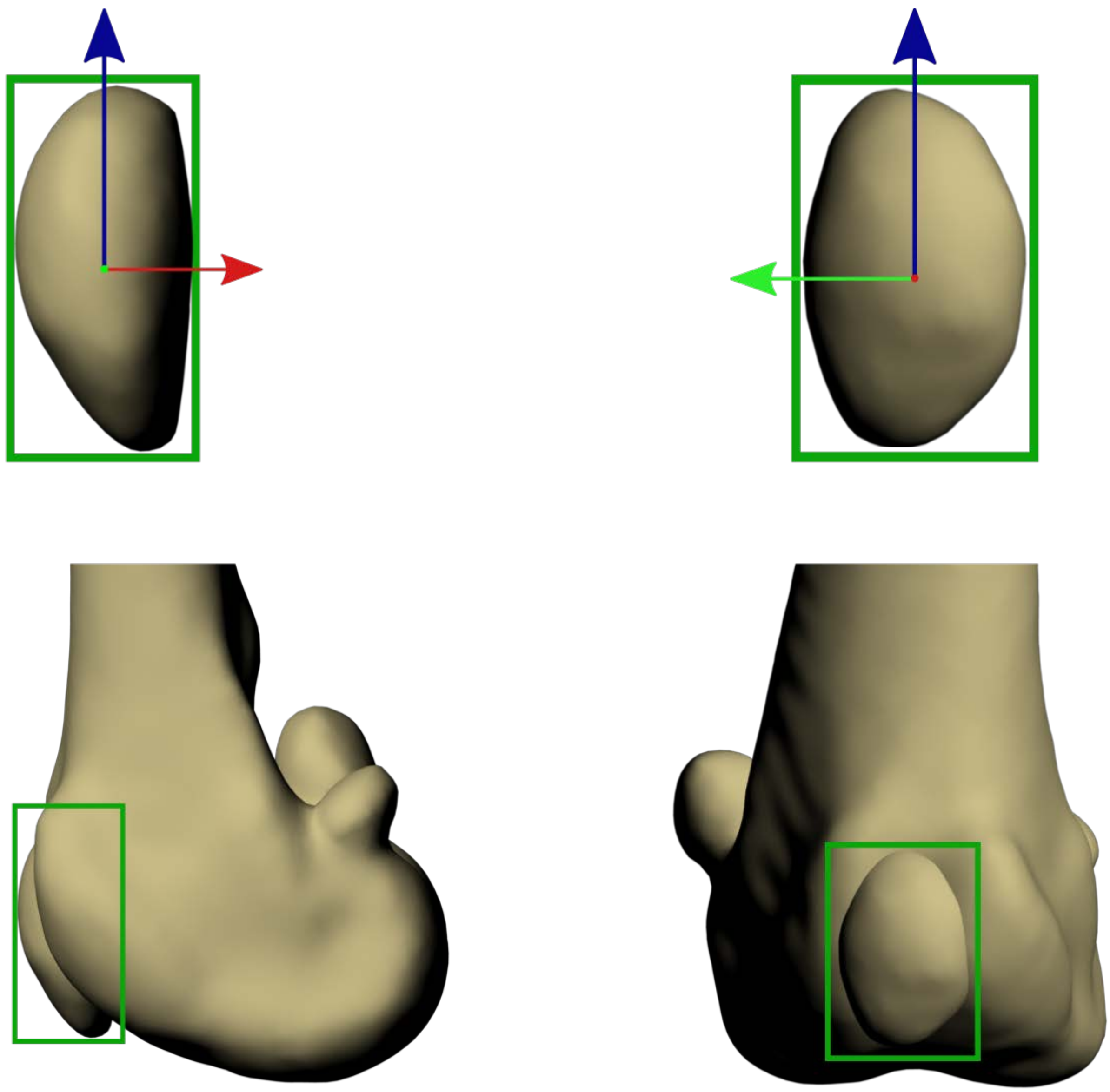

Figure S3 The patella was placed in vertical position, with the *basis patellae* and the *apex patellae* approximately aligned in the sagittal plane. In the frontal plane, the patella was centered in the sphere (see Fig. S2). Then, the patella was moved towards the sphere (in both sagittal and dorsoventral planes) until the vertical crest of the *facies articularis* of the patella touched the sphere. A cube that enclosed the patella was used to find the patella's midpoint.

Coordinate systems were aligned to the axes of the global coordinate system [x (red, positive direction points caudally, y (green, positive direction points medially) and z (blue, positive direction points upwards)]. Green, and red dots indicate that those axes point into the picture.

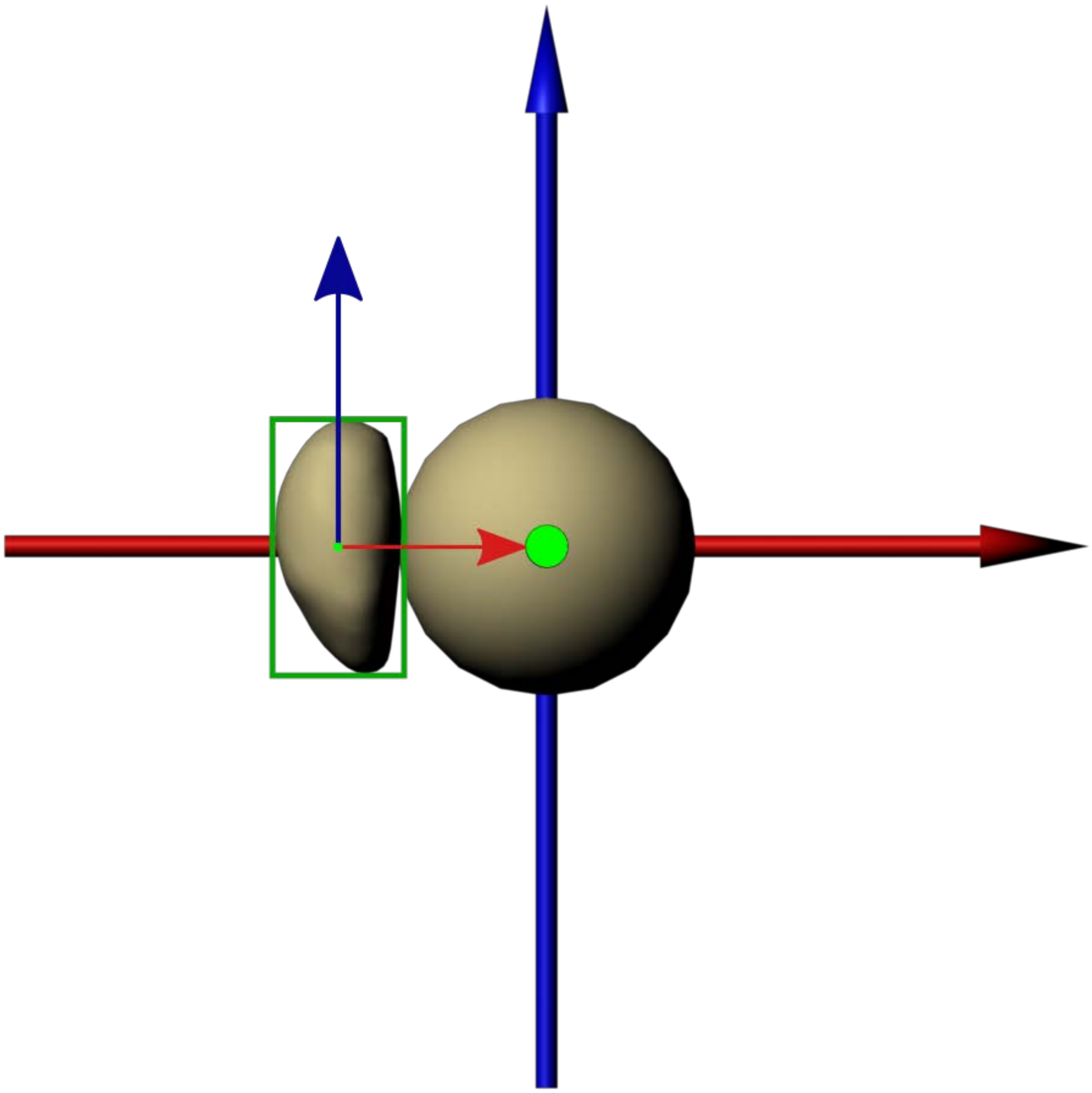

Figure S4 The vertical crest of the *facies articularis* of the patella was moved until it touched the sphere (compared to Figures S2 and S3, the femur was made invisible in this render). Patellar coordinate system was then aligned with the patellofemoral joint. Green dots indicate that those axes point into the picture.
